# A Non-Invasive Hydration Monitoring Technique Using Microwave Transmission and Data-Driven Approaches

**DOI:** 10.3390/s22072536

**Published:** 2022-03-25

**Authors:** Deepesh Agarwal, Philip Randall, Zachary White, Bayleigh Bisnette, Jenalee Dickson, Cross Allen, Faraz Chamani, Punit Prakash, Carl Ade, Balasubramaniam Natarajan

**Affiliations:** 1 Department of Electrical and Computer Engineering, Kansas State University, Manhattan, KS 66506, USA; deepesh@ksu.edu (D.A.); philip12@ksu.edu (P.R.); bisnbayl@ksu.edu (B.B.); jenaleed@ksu.edu (J.D.); cjallen383@ksu.edu (C.A.); faraz@ksu.edu (F.C.); bala@ksu.edu (B.N.); 2 Department of Kinesiology, Kansas State University, Manhattan, KS 66506, USA; zjwhite@ksu.edu (Z.W.); cade@ksu.edu (C.A.)

**Keywords:** hydration monitoring, non-invasive, microwave transmission, regression analysis, hypohydration and euhydration

## Abstract

Dehydration in the human body arises due to inadequate replenishment of fluids. An appropriate level of hydration is essential for optimal functioning of the human body, and complications ranging from mild discomfort to, in severe cases, death, could result from a neglected imbalance in fluid levels. Regular and accurate monitoring of hydration status can provide meaningful information for people operating in stressful environmental conditions, such as athletes, military professionals and the elderly. In this study, we propose a non-invasive hydration monitoring technique employing non-ionizing electromagnetic power in the microwave band to estimate the changes in the water content of the whole body. Specifically, we investigate changes in the attenuation coefficient in the frequency range 2–3.5 GHz between a pair of planar antennas positioned across a participant’s arm during various states of hydration. Twenty healthy young adults (10M, 10F) underwent controlled hypohydration and euhydration control bouts. The attenuation coefficient was compared among trials and used to predict changes in body mass. Volunteers lost 1.50±0.44% and 0.49±0.54% body mass during hypohydration and euhydration, respectively. The microwave transmission-based attenuation coefficient (2–3.5 GHz) was accurate in predicting changes in hydration status. The corresponding regression analysis demonstrates that building separate estimation models for dehydration and rehydration phases offer better predictive performance (88%) relative to a common model for both the phases (76%).

## 1. Introduction

Accurate monitoring of whole-body hydration is important for many categories of end-users, including the elderly, athletes, the military, and industrial workers, operating in stressful environmental conditions [1]. Thirst provides a source of feedback to individuals; however, this bodily sensation can be less pronounced or completely absent. It may also be a delayed indicator of hydration level, even when fully unimpaired since the body cannot accurately assess if fluid homeostasis has been restored until minutes after ingestion [2]. Hence, it is difficult for individuals to assess their hydration status at lower levels with perceptions of thirst alone. As little as a 1% decrease in bodyweight due to fluid loss can result in impaired cognitive function, increased anxiety levels, and fatigue during physical activity [3]. In athletes, it is well established that a decrease in total body water even by a meager amount of 5% or less leads to a 6–48% decline in physical work capacity, thereby adversely influencing their exercise performance. Importantly, multiple studies have shown that as little as 1% decrease in body mass, a surrogate for changes in total body water, is associated with at 6% decrease in exercise performance [4,5]. In athletic populations, including American football, rowing, soccer, and basketball, training and competition has been shown to elicit a similar 1–2% decrease in body mass, highlighting the critical need to evaluate hydration status during normal athletic activities. However, to date, there remains a paucity of options to evaluate hydration status quickly and accurately in these and other populations at risk of dehydration.

Some prior studies have investigated electromagnetic sensing methods for monitoring skin hydration. One of the early works employs a terahertz (THz) time-domain spectroscopy technique to determine reflectance, which in turn characterizes the variation in water content [6]. This work is motivated by the strong correlation between THz radiation and water content. Schiavoni et al. have proposed the use of a time-domain reflectometry system relying on measures of dielectric permittivity and electrical conductivity variations of the skin to examine the hydration status [7]. This is based on the idea that changes in metabolic activity and composition of the skin lead to modifications in its electrical properties. Another study by Rizwan et al. introduces Galvanic Skin Response (GSR) as a surrogate measure of hydration levels [8]. The hydration status is distinguished as hydrated vs. dehydrated in various body postures such as sitting and standing. However, all these studies consider hydration assessment at the skin and, thus, do not provide an assessment of whole-body hydration changes, which is the focus of the present study.

As recently reviewed by Garrett et al., there are few reliable and accurate methods for non-invasive measurement of whole-body water content [9]. The adverse effects of both mild and severe dehydration could be mitigated, or possibly avoided, in these vulnerable groups with a robust, non-invasive system that can rapidly detect a 2% or smaller change in body weight due to fluid imbalances. When detected early, mild dehydration can easily be treated by administering food and drink to the affected individual. Overhydration could also be prevented with this system by providing an estimate of how much fluid must be replaced to attain the ideal hydration window. The ability to accurately monitor hydration status in real-time would ensure the optimal performance and well-being of people that are susceptible to frequent bouts of dehydration, whether it is due to the environment, disease, or an inability to communicate (such as infants).

In this context, early dehydration monitoring systems must be able to detect a <2% change in whole-body water content, as this is a threshold at which observable reductions in performance have been observed. The electrical properties of several tissues are dominated by the properties of water, given that water constitutes ∼60–70% of many tissue types. Thus, detecting changes in electrical properties of tissues, or parameters related to these properties, have been proposed as an indirect method for tracking changes in hydration. Moran et al. used a pair of 916 MHz antennas positioned on either side of the subject’s arm to measure the magnitude of the electromagnetic attenuation coefficient [10]. Changes in the attenuation coefficient were measured in subjects prior to and after conducting 30 min exercise in heat stress and were shown to be predictive of weight loss, attributed to water loss. Garrett et al. investigated a similar approach using a broadband pair of antennas but extended the technique to include the complex valued attenuation coefficient [9,11]. They further proposed a parametric model for estimating the permittivity of the effective medium between the antennas and investigated the relationship between this parameter and hydration changes following exercise in college athletes. A limitation of the prior studies is the lack of euhydration controls and the results are not disaggregated by sex.

Several recent studies have exploited data-driven and statistical machine learning-based methods for estimation of hydration status. The relationship between hydration and cardiovascular responses to orthostatic changes is leveraged to assess hydration status by framing the task as a binary classification problem [12]. Alvaraz et al. employ Support Vector Machines (SVM) and k-means to implement a three-stage dehydration protocol for athletes using electrocardiograph signals [13]. Variable frequency complex demodulation is used to track changes in amplitudes of Photoplethysmographic (PPG) signals and detect dehydration by SVM with a radial basis function kernel [14]. However, the predictive performance of the aforementioned methodologies for <2% acute changes in hydration is not encouraging enough to be deployed for field applications.

Here, we report a study on investigating changes in the attenuation coefficient between a pair of planar antennas positioned across a subject’s arm during various states of hydration. Changes in the electromagnetic attenuation coefficient in the frequency range 2–3.5 GHz were analyzed to assess their potential for predicting changes in body weight over 30 min periods during periods of heat stress (hypohydration), rest (control euhydration), or recovery following heat stress (rehydration). Data were gathered from 20 subjects (10 male, 10 female) ranging 23±4 years in age. A hydration assessment was formulated as a regression problem with percentage change in body weight as the target and features derived from attenuation coefficient in the frequency range 2–3.5 GHz as predictors. The results were then disaggregated by sex. As detailed in the following sections, this work illustrates the technical feasibility of a microwave transmission-based technique, and accompanying data analysis approaches, to predict <2% acute changes in body hydration. In summary, the capability of predicting <2% acute changes in body hydration with customized data-driven models for males and females during dehydration and rehydration phases address the key limitations of the current state-of-the-art.

## 2. Materials and Methods

We performed an initial set of experiments using agar phantom gels made with varying water content. The primary goals of conducting these pilot experiments included: (i) to test the hypothesis that changes in gel water content could be captured using the proposed electromagnetic measurements, i.e., attenuation coefficient between a pair of planar antennas; (ii) to identify the frequency bands in electromagnetic measurements that possess discriminatory power to represent changes in water content. Consequently, the study was extended to human participants as per the protocol illustrated in Section 2.2.

### 2.1. Experiment 1—Gel Studies

**Figure 1:**
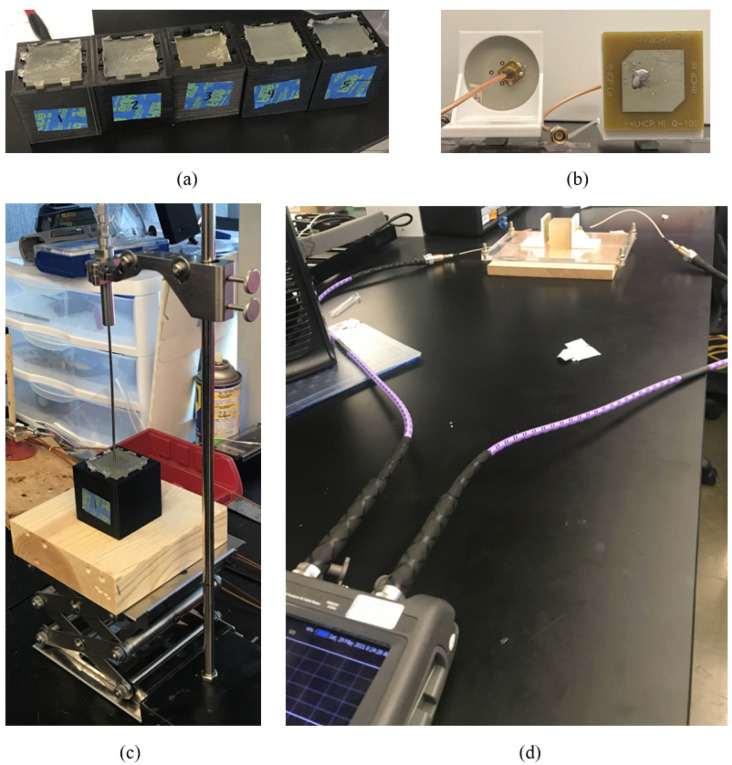
Setup for the gel studies: (**a**) five agar gels with varying water content, (**b**) close-up image of the antennas, (**c**) measurement of dielectric properties, and (**d**) capturing of electromagnetic measurements.

In order to test the hypothesis that changes in gel water content can be captured using the proposed electromagnetic measurements, five agar gels were formed. The reference gel was made with approximately 3 g agar (Becton, Dickinson and Company, Franklin Lakes, NJ, USA), 34.5 g sugar, 0.375 g of NaCl, and 112 mL distilled water. While keeping the rest of the ingredients constant, the water content was varied to make four more gels with 106, 110, 115, and 118 ml water (−5%, −2%, +2% and +5% compared to the reference gel, respectively). These gels were allowed to set and were subsequently tested for their dielectric properties and electromagnetic measurements. The complex-value relative permittivity of the gels was measured using a Keysight 85070E dielectric probe kit (Keysight Technologies, Inc., Santa Rosa, CA, USA) [15]. Electromagnetic transmission coefficient (S21) was measured between a pair of 2.4 GHz circular-polarized patch antennas [16] placed around 7 cm apart, with the gel sample positioned in between the antennas. The measured quantity is S21 magnitudes and the corresponding setup is shown in Figure 1. Five agar gels with varying water content are shown in Figure 1a. The measurement of dielectric and electromagnetic properties are illustrated in Figure 1b,c, respectively.

The frequency bands possessing discriminatory power were selected based on two-sample t-tests for several frequency bands across data from all the gel samples with varying water content (i.e., ±5% and ±2%). The null hypotheses for these tests is that measurements corresponding to a particular frequency band in the reference gel and the gels with varying water content belong to independent random samples from normal distributions with equal means. These tests were carried out individually for all the four gels: ±5% and ±2%. Consequently, four bands possessing the highest discriminatory power were selected and the corresponding *p*-values are shown in Table 1. The selected frequency bands are: 2.0–2.7 GHz (F1); 2.7–3.4 GHz (F2); 2.44–2.47 GHz (F3); and 2.2–2.4 GHz (F4).

It can be seen in Table 1 that *p*-values are less than 0.05 for all the gels across all frequency bands, indicating a statistically significant difference in electromagnetic measurements corresponding to selected frequency bands between the test gels and the reference gel. Thus, the selected frequency bands possess discriminatory power to represent changes in water content.

The dielectric properties of the gels are reported as complex-valued relative permittivity, $\epsilon_{r}^{*}$ shown in Equation (1). Here, $\epsilon_{r}^{\prime }$ and $\epsilon_{r}^{\prime \prime }$ represent the real and imaginary parts of the complex-valued relative permittivity $\epsilon_{r}^{*}$.

(1) $$\epsilon_{r}^{*}=\epsilon_{r}^{\prime }-j\epsilon_{r}^{\prime \prime }$$

**Figure 2:**
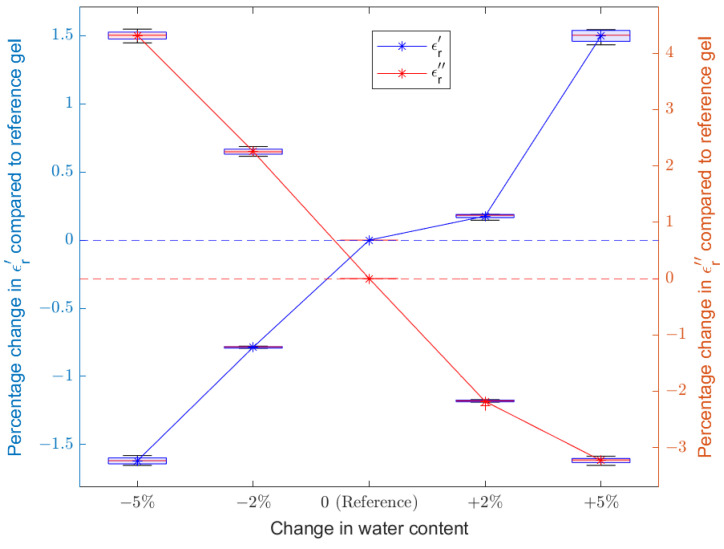
Plot of changes in dielectric properties of agar phantom gels with increasing water content.

**Figure 3:**
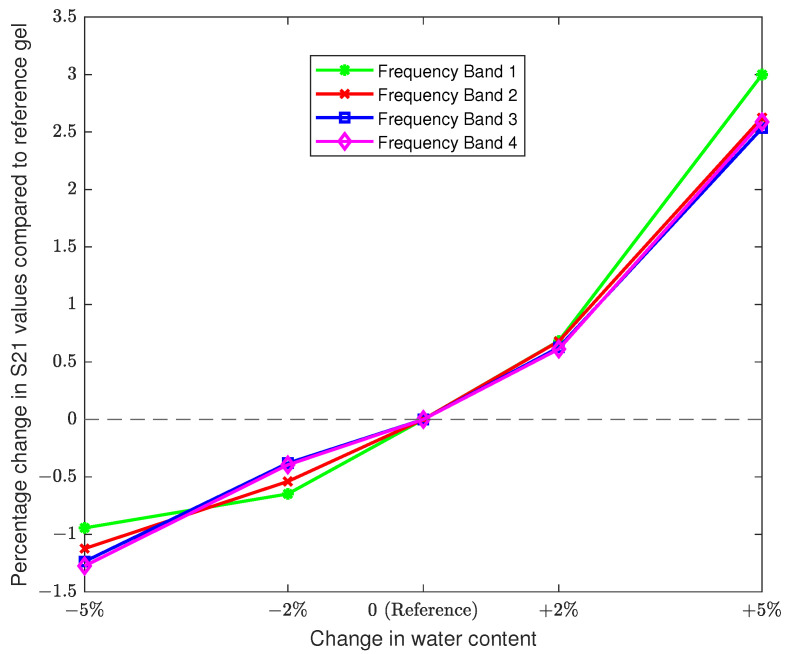
Plot of changes in electromagnetic transmission coefficient (S21) measurements of agar phantom gels with increasing water content.

The complex-valued relative permittivity of the reference gel was 63.36−j16.95 at 2.45 GHz. The percentage changes in values of $\epsilon_{r}^{\prime }$ and $\epsilon_{r}^{\prime \prime }$ are reported over the band of 2.4–2.5 GHz and are summarized in Table 2. The corresponding plots for dielectric properties and electromagnetic transmission coefficient (S21) measurements are shown in Figure 2 and Figure 3, respectively. It can be seen that the changes in the mean of S21 magnitudes (with respect to the reference gel) in all the four frequency bands increase with increasing water content, ranging roughly between −1.5% and +3%. This validates our hypothesis that short term changes in body weight attributed to water loss/gain can be captured using the proposed electromagnetic measurements. As the next step, we extended this study to human participants as detailed in the forthcoming subsection.

### 2.2. Experiment 2—Extension to Human Participants

Twenty recreationally active participants, men (n=10) and women (n=10) (age 23±4 years (mean ± SD); height 172.6±8.5 cm; mass 72.0±15.7 kg), volunteered to participate in the current investigation. Inclusion criteria included meeting the American College of Sports Medicine’s current aerobic physical activity guidelines (>150 min per week of moderate physical activity or >75 min of vigorous physical activity), with experience exercising in a warm environment. In addition, participants could have no history of heat-related illness. Participants were excluded based on a prior diagnosis of cardiovascular disease or traditional cardiovascular risk factors, including hypertension, current smoker, hyperlipidemia and elevated cholesterol, diabetes, or metabolic syndrome. Participants were also excluded if they had sickle cell anemia, a history of heat-related illness or currently had risk factors for heat-related illness, including preceding viral infection, dehydration, fatigue, lack of sleep, or poor physical fitness, were currently taking antihistamines, anti-nausea, and pseudoephedrine. All experimental procedures and methods were approved by the Institutional Review Board of Kansas State University and conformed to the standards set forth by the Declaration of Helsinki. Prior to data collection, all participants gave verbal and written informed consent.

#### 2.2.1. Protocol

**Figure 4:**
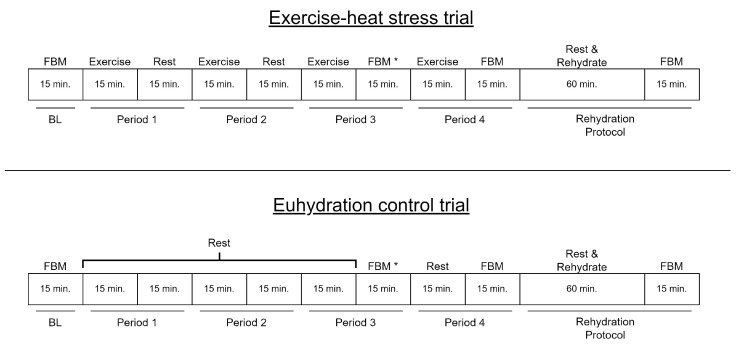
Timeline of events in each treatment condition. Fluid balance measurements (FBM): nude body weight, urine specific gravity, hematocrit and S21 measurement; * Period 3 FBM: urine specific gravity was not assessed. BL: baseline.

A randomized cross-over study design that was utilized consisted of two experimental visits spaced at least 7 days apart. All trials were performed at approximately the same time of day. Prior to each visit, participants consumed 3L of water 24 h prior to the experiment with a 2 h fast. On the day of each experimental visit, the participant provided a urine sample, and all measurements were performed. The experimental trial consisted of either an exercise-heat stress to induce hypohydration corresponding to a body water deficit of <3% of body mass loss or a euhydration control, as described in Figure 4. The exercise-heat stress trial consisted of intermittent cycle exercise consisting of 30-min cycles consisting of a 15-min moderate intensity intermittent treadmill or stationary bicycle exercise period at 50–70% of age-predicted maximal heart rate and a 15-min measurement period, with a total of 4 exercise-rest periods performed. The exercise room temperature was maintained between 26 and 32 °C and 40 and 80% relative humidity. Immediately following the exercise-heat stress, a rehydration protocol was performed and consisted of participants sitting in a temperate room (∼20 °C) and consuming 1 L of sodium/electrolyte beverage (potassium, 139 mg; sodium, 444 mg; total carbohydrates, 61 g) over 60 min followed by a final measurement period (RHY). Euhydration control was aimed at maintaining a constant hydration status and consisted of four 15-min resting seated periods interspersed with 15-min measurement periods.

At the baseline and each measurement period, fluid balance was determined via nude body weight, urine specific gravity, and hematocrit. Nude body weight (NBW; kg) was assessed using a digital platform scale (Health o meter Professional; Model 349KLX) (coefficient of variation = 0.06%). Instructions were to remove clothes, remove any access sweat with a given a towel, and step onto the scale. Total nude body mass and total body water loss were considered equivalent (1 mL = 1g) after correction for respiratory water loss [17,18]. Urine specific gravity was determined from urine samples. Participants were given a sterile urine container and given instructions on how to provide a mid-stream urine sample. ∼1 mL of sample was analyzed with a digital palette refractometer (Atago Co., Ltd., Tokyo, Japan) [19]. The refractometer was calibrated before each analysis, and absolute USG change from baseline ($USG_{BL}$) to end of exercise ($USG_{i}$) was used for analyses. Hematocrit (HCT) was determined from a blood sample obtained via a finger stick. Briefly, the finger was sterilized with isopropyl alcohol pad, allowed to air dry, and punctured with a safety lancet. To promote bleeding, hands were warmed with a heating pad prior to puncture or light pressure was applied just below the puncture site. Blood was collected in heparinized capillary tubes (I.D., 1.1–1.2 mm; length, 75 mm; wall, 0.2±0.02 mm) and promptly centrifuged for 5 minutes. After separation, HCT was calculated as the length of the RBC layer (mm)/total length of the sample (mm). HCT was analyzed as percent plasma volume change from baseline, calculated as shown in Equation (2).

(2) $$HCT=\frac{100}{100-HCT_{i}}\frac{(HCT_{i}-HCT_{BL})100}{HCT_{BL}}$$

**Figure 5:**
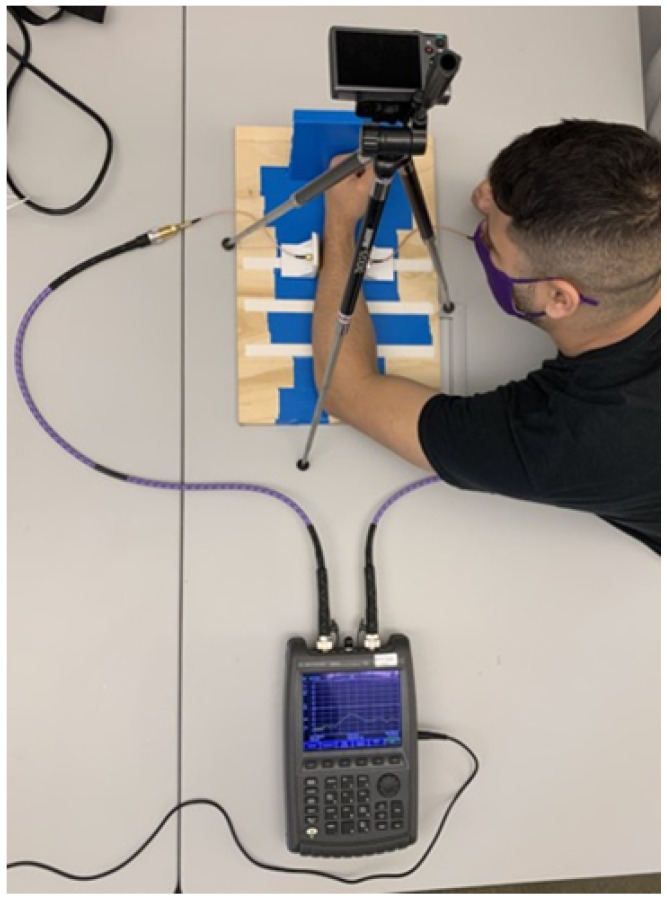
Experimental setup used to obtain S21 measurements from the participant.

The transmission coefficient across the subject’s wrist was taken with a S21 measurement using the Keysight FieldFox N9923A portable vector network analyzer (Keysight Technologies, Inc., Santa Rosa, CA, USA) [20]. The analyzer is connected to two microstrip patch antennas optimized for operation at 2.4 GHz and placed around 7 cm apart. Figure 5 depicts the full setup with the forearm placement used for an S21 measurement. The frequency range was set between 1 and 4 GHz on the network analyzer, and calibration was performed each time before operating to ensure a consistent baseline. An adjustable wooden backboard was used as a reference for arm placement, and a camera mounted on a tripod was placed over the antennas to capture an image of each measurement for reference in case some data were found to be inconsistent. An S21 measurement was taken four times during each trial: “Baseline”, “After Period 3”, “After Period 4”, and “After Rehydration”. The subjects were asked to place their left wrist between the antennas with their elbow centered, and the antennas were situated to have a few millimeters of clearance between the conductor and the skin. Once the subject’s arm was in position, an overhead photo was taken of the arm and the S21 data were collected. The photos were taken to backtrack any inconsistencies in data corresponding to arm position for specific participants during statistical analyses steps. The S21 measurement was repeated three times back-to-back for each period to ensure consistency in the data, especially with the possibility of movement artifacts, resulting in twelve total measurement files per experiment.

#### 2.2.2. Statistical Analyses of Hydration Indicators

**Figure 6:**
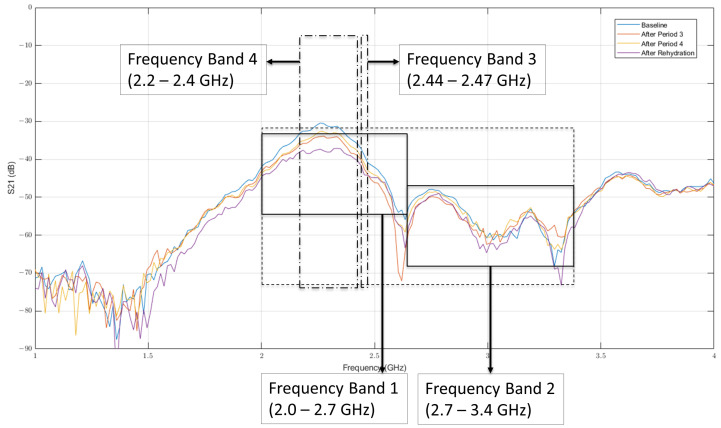
Sample plot of S21 magnitudes for one participant during different instances.

The first step is to identify the parameter that characterizes hydration in the human body. During the experiments, three potential parameters, namely, body weight (*w*), Plasma Volume (PV) and Urine Specific Gravity (USG), were measured at regular intervals as described in Section 2.2.1. This was achieved by performing two-sample t-tests for all the candidate parameters across the set of measurements obtained from “exercise” and “control” trials. The test decision values and *p*-values for the null hypothesis that measurements in “exercise” and “control” groups belong to independent random samples from normal distributions with equal means were evaluated for all three parameters at instances “After Period 3”, “After Period 4”, and “After Rehydration”. The alternate hypothesis in these tests is that the measurements in “exercise” and “control” conditions belong to populations with unequal means. The two-sample t-tests were performed using the Statistics toolbox in MATLAB. In this article, the problem of predicting small changes (up to ∼2% of body weight) in body hydration using non-invasive approaches was modeled as a regression problem. The percentage change in value of the parameter characterizing hydration is used as a target variable and the mean of S21 magnitudes of the selected frequency bands in electromagnetic data are used as predictors. A sample plot of S21 magnitudes for one participant during different instances is shown in Figure 6.

The first two frequency bands are selected to be the wider ones between 2.0–2.7 GHz and 2.7–3.4 GHz. These are the regions where noise is relatively low (unlike the region 1.0–1.75 GHz), and there is a visual difference between the plots corresponding to different time instances. The third frequency band is a narrow band (2.44–2.47 GHz) centered around the tuning frequency of the antenna. The last frequency band is selected as 2.2–2.4 GHz, i.e., the region in which the values of S21 magnitudes reach their corresponding peaks. These frequency bands are the same as the ones selected via conducting two-sample *t*-tests for gel studies (Table 1). The discriminatory power of these bands for the study of human participants was again verified using similar *t*-tests for the measurement times “After Period 3”, “After Period 4”, and “After Rehydration”. The corresponding *p*-values are summarized in Table 3. It can be seen in Table 3 that *p*-values are less than 0.05 for all the measurement times across all frequency bands, indicating a statistically significant difference in electromagnetic measurements corresponding to selected frequency bands between these measurement times and the “baseline” measurement. The means of S21 magnitudes in these four frequency bands are used as predictor variables for the regression problem.

The regression models are built using three different techniques, namely, Linear Regression (LR), Decision Tree Regression (DTR), and Support Vector Regression (SVR), and these are compared to identify the one which best fits the data, based on their mean squared error (MSE) and R-squared values. These regression techniques are chosen considering their well-recognized advantages. The LR models are easy to implement, and the corresponding linear equations are fairly easy to understand and interpret [21]. DTR is a non-parametric method having no assumptions about model structure, is easier to implement and does not require much pre-processing efforts [22]. SVR is a kernel-based technique allowing to work with arbitrary large feature space and offers good generalization performance [23]. Moreover, these methods have been extensively used in many bioengineering applications including classification of cardiac diseases [24], cardiac abnormalities [25], diagnosis of diabetes [26], detection of Alzheimer’s disease [27], classification of metabolic diseases [28], and COVID-19 diagnosis [29].

Prior to building regression models, the correlation analysis was performed for each pair of target and predictor variables to investigate the strength of linear relationship between them. The calculations corresponding to percentage changes in *w*, PV and USG at an instant *t*, given by $\Delta w_{t}$, $\Delta PV_{t}$ and $\Delta USG_{t}$, are performed as shown in Equations (3)–(5), respectively. Here, $w_{ref}$, $HCT_{ref}$ and $USG_{ref}$ are the corresponding reference measurements taken before starting any protocol, and $w_{t}$, $PV_{t}$ and $USG_{t}$ are the corresponding measurements at time *t*.

(3) $$\Delta w_{t}=\frac{w_{t}-w_{ref}}{w_{ref}}\times 100$$

(4) $$\Delta PV_{t}=\frac{100}{100-HCT_{ref}}\times \frac{(HCT_{ref}-HCT_{t})100}{HCT_{t}}$$

(5) $$\Delta USG_{t}=\frac{USG_{ref}-USG_{t}}{USG_{ref}}\times 100$$

## 3. Results

### 3.1. Hydration Indicators

**Figure 7:**
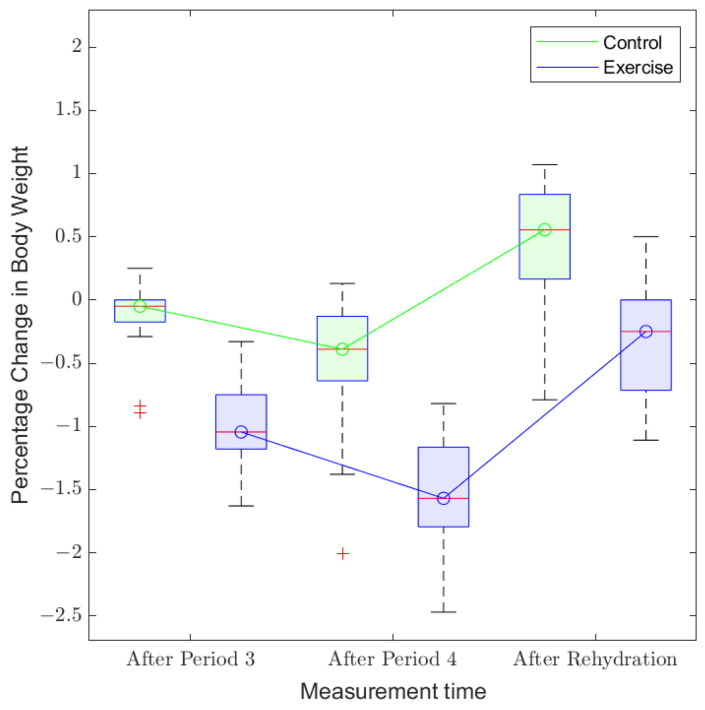
Summary of variations in percentage changes in body weight over different time intervals for the participants.

**Figure 8:**
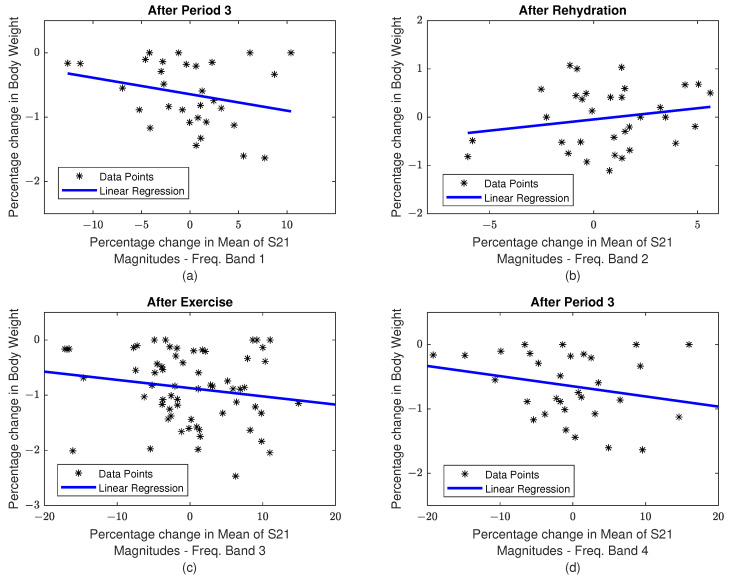
Correlation plots for selected cases. Percentage changes in (**a**): body weight vs. mean of S21 magnitudes in frequency band 1 during the measurement time “After Period 3”; (**b**): body weight vs. mean of S21 magnitudes in frequency band 2 during the measurement time “After Rehydration”; (**c**): body weight vs. mean of S21 magnitudes in frequency band 3 during the measurement time “After Exercise”; (**d**): body weight vs. mean of S21 magnitudes in frequency band 4 during the measurement time “After Period 3”.

In order to identify the parameter that best characterizes hydration in the human body, two-sample t-tests were conducted for all the candidate parameters across the set of measurements obtained from “exercise” and “control” trials. The corresponding results are tabulated in Table 4. A plot showing a summary of variations in percentage changes in body weight over different time intervals for the participants during both “exercise” and “control” treatments is presented in Figure 7. The correlation analysis is performed between each pair of target and predictor variables to investigate the strength of the linear relationship between them. In this analysis, there is only one target variable, i.e., $\Delta w_{t}$. The correlation coefficients corresponding to different predictor variables during all measurement times are tabulated in Table 5. The correlation plots for selected cases are presented in Figure 8. Here, the measurements during “After Exercise” includes a combination of measurements captured during “After Period 3” and “After Period 4” periods.

### 3.2. Hydration Prediction Models

We perform two types of analyses in this work: (i) general analysis (considering male and female participants together) and (ii): sex-specific analysis. For the general analysis, we consider two cases: (1) building separate regression models for dehydration and rehydration phases; and (2) a common model for both dehydration and rehydration phases. Here, the instance “Dehydration Phase” includes the measurements at the end of exercise phase, i.e., combines the measurements captured during “After Period 3” and “After Period 4”. In both these cases, regression analysis is performed by building two sets of models—one using $\Delta F1_{t}$, $\Delta F2_{t}$, $\Delta F3_{t}$, and $\Delta F4_{t}$ as predictors and another using $\Delta PV_{t}$ and $\Delta USG_{t}$ as predictors. The resulting values of MSE, ordinary R-squared, adjusted R-squared, and predictive performance for cases 1 and 2 are indicated in Table 6 and Table 7, respectively. The regression models with $\Delta \bar{F1_{t}}$, $\Delta \bar{F2_{t}}$, $\Delta \bar{F3_{t}}$, and $\Delta \bar{F4_{t}}$ as predictors are considered for sex-specific analysis as their performance was observed to be better than those built using $\Delta \bar{PV_{t}}$ and $\Delta \bar{USG_{t}}$ as predictors. We consider two different cases for sex-specific analysis: (1) using the models originally trained on the entire data (both male and female participants); and (2) training separate models using data from male and female participants. All the performance metrics, including MSE, ordinary R-squared, adjusted R-squared, and predictive performance are computed similar to that in the general analysis. The results for cases 1 and 2 are presented in Table 8 and Table 9, respectively.

## 4. Discussion

The parameter that best characterizes hydration in the human body is identified from Table 4. From Table 4, it can be seen that the test decision values corresponding to $\Delta w_{t}$ are 1 and the *p*-values are less than 0.05 for all the measurement times. This indicates that the difference in measurements from “exercise” and “control” groups is statistically significant. On the other hand, the test decision values corresponding to $\Delta PV_{t}$ and $\Delta USG_{t}$ are 0 for some of the measurement times, indicating that the difference in measurements from “exercise” and “control” groups is statistically not significant during all the time intervals. Thus, the parameter $w_{t}$ is chosen to be representative of hydration in the human body and is treated as target variable in the regression analysis demonstrated in this article.

The variations in percentage changes in body weight over different time intervals for the participants during both “exercise” and “control” treatments are demonstrated in Figure 7. It can be observed that the body weight decreases slightly up to “After Period 4”, when the participant is subjected to physical stress and it increases again during the process of rehydration, when the participant is directed to consume a pre-defined amount of water. Moreover, the extent of change in body weight is higher for the “exercise” treatment, as compared to that for the “control”. This is intuitive in that the participant encounters a higher degree of physical load during the “exercise” phase.

The next step is to perform a correlation analysis between each pair of targets (i.e., $\Delta w_{t}$) and predictor variables in order to investigate the strength of the linear relationship between them. From Table 5 and Figure 8, it can be seen that none of the combinations of target and predictor variables exhibits a strong linear relationship individually. This suggests the need to perform regression analysis by using multiple predictor variables and explore other methods of building regression models, in addition to the linear regression approach. Here, the percentage change in the mean of S21 magnitudes corresponding to different frequency bands is calculated as shown in Equation (6). Here, $\bar{Fx_{ref}}$ is the reference measurements for frequency band *x* taken before starting any protocol and $\bar{Fx_{t}}$ is the corresponding measurement at time *t*.

(6) $$\Delta \bar{Fx_{t}}=\frac{\bar{Fx_{t}}-\bar{Fx_{ref}}}{\bar{Fx_{ref}}}\times 100$$

In general analysis (considering male and female participants together), we build two sets of models—one using $\Delta \bar{F1_{t}}$, $\Delta \bar{F2_{t}}$, $\Delta \bar{F3_{t}}$, and $\Delta \bar{F4_{t}}$ as predictors and another using $\Delta \bar{PV_{t}}$ and $\Delta \bar{USG_{t}}$ as predictors. These models are built for two different cases: (1) separate regression models for dehydration and rehydration phases; and (2) common model for both dehydration and rehydration phases. The performance is evaluated using the metrics: MSE, ordinary R-squared, adjusted R-squared, and predictive performance. The adjusted R-squared statistic is computed as it helps to mitigate the overfitting issue in ordinary R-squared values by penalizing additional independent variables added to the model. The predictive performance values are calculated using Equation (7). Here, $A_{i}$ and $P_{i}$ are actual and predicted values, respectively, and *n* is the total number of observations.

(7) $$\mathrm{Predictive}\;\mathrm{Performance}=100-\frac{100}{n}\sum_{i=1}^{n}\left|\frac{A_{i}-P_{i}}{A_{i}}\right|$$

The results for cases 1 and 2 of the general analysis are tabulated in Table 6 and Table 7, respectively. Firstly, it is established that $\Delta w_{t}$ (representative of hydration in the human body) can be predicted using measurements obtained from the proposed electromagnetic assessment technique. Secondly, it is observed that the regression models built using data obtained from the proposed electromagnetic assessment technique as predictors provide a better fit than those built using PV and USG as predictors. The best performance is exhibited by regression models built using the SVR technique. Additionally, it can be seen that training separate models for dehydration and rehydration phases gives better predictive performance (up to 89%) and better fits (adjusted R^2^ value of 0.82) as compared to having a common model trained using the entire data (up to 77% and adjusted R^2^ value of 0.72).

**Figure 9:**
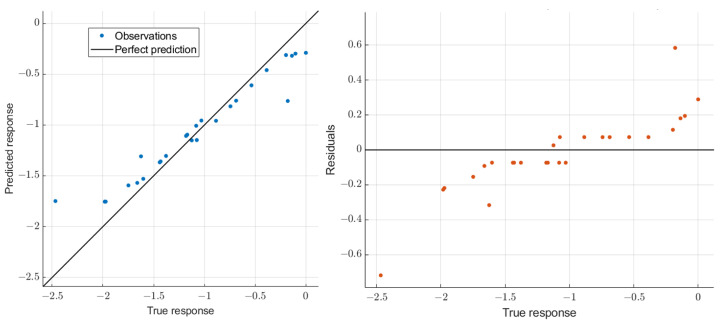
Sample plots of true response vs. predicted response and residuals for predictions with SVR for male participants in sex-specific Analysis—Case 2.

We build the regression models with $\Delta \bar{F1_{t}}$, $\Delta \bar{F2_{t}}$, $\Delta \bar{F3_{t}}$, and $\Delta \bar{F4_{t}}$ as predictors for sex-specific analysis as their performance was observed to be better than those built using $\Delta \bar{PV_{t}}$ and $\Delta \bar{USG_{t}}$ as predictors. The models are built and their performance is analyzed for two different cases: (1) using the models originally trained on the entire data (both male and female participants); and (2) training separate models using data from male and female participants. The corresponding results are presented in Table 8 and Table 9, respectively. These results indicate that training separate models using data from male and female participants provides better predictive performance (up to 92%) as compared to using a common model across both genders (up to 89%). Finally, it is observed that the models in the dehydration phase provide a better predictive performance as compared to those in the rehydration phase. The sample plots of true response vs. predicted response and residuals for predictions with SVR for male participants in the sex-specific Analysis—Case 2 are shown in Figure 9.

## 5. Conclusions

In this work, we have proposed a non-invasive hydration monitoring technique employing non-ionizing electromagnetic power in the microwave band to estimate the changes in whole-body water content. Specifically, we investigate changes in the attenuation coefficient in the frequency range 2–3.5 GHz between a pair of planar antennas positioned across a participant’s arm during various states of hydration. Initially, we hypothesize that the changes in water content can be captured using the proposed electromagnetic measurements, i.e., attenuation coefficient between a pair of planar antennas. We performed initial studies with five agar phantom gels of different water content made in laboratory. After validating our hypothesis using experiments with agar gels, we extended the study to human participants. Firstly, we establish that body weight is an accurate representation of the hydration status as compared to other measures such as PV and USG. Secondly, we formulate a regression problem with a percentage change in body weight as target and electromagnetic measurements as predictors. The corresponding data analysis demonstrates the ability to predict <2% acute changes in whole-body hydration. It is observed that building separate estimation models for dehydration and rehydration phases provide better predictive performance (88%) as opposed to having a common model for both the phases (76%).

## Figures and Tables

**Table 1:** *p*-values of two-sample *t*-tests for selection of frequency bands having discriminatory power in gel studies.

Frequency Bands	Water Content			
	−5%	−2%	+2%	+5%
F1 (2.0–2.7 GHz)	$1.89\times 10^{-2}$	$2.47\times 10^{-6}$	$4.10\times 10^{-6}$	$4.24\times 10^{-4}$
F2 (2.7–3.4 GHz)	$3.73\times 10^{-4}$	$2.36\times 10^{-7}$	$1.06\times 10^{-6}$	$6.10\times 10^{-3}$
F3 (2.44–2.47 GHz)	$4.37\times 10^{-12}$	$8.86\times 10^{-8}$	$2.25\times 10^{-7}$	$1.11\times 10^{-6}$
F4 (2.2–2.4 GHz)	$3.46\times 10^{-2}$	$1.20\times 10^{-3}$	$3.97\times 10^{-8}$	$8.15\times 10^{-5}$

**Table 2:** Percentage changes in dielectric properties and electromagnetic transmission coefficient (S21) measurements of agar phantom gels with respect to the reference gel.

		Water Content Change Relative to Reference gel			
		−5%	−2%	+2%	+5%
**Measures**	$\epsilon_{r}^{\prime }$	−1.62%	−0.78%	0.18%	1.50%
	$\epsilon_{r}^{\prime \prime }$	4.31%	2.25%	−2.18%	−3.23%
	S21-F1	−0.95%	−0.65%	0.68%	2.99%
	S21-F2	−1.12%	−0.54%	0.68%	2.62%
	S21-F3	−1.24%	−0.38%	0.63%	2.53%
	S21-F4	−1.28%	−0.40%	0.61%	2.59%

**Table 3:** The *p*-values of two-sample t-tests for selection of frequency bands having discriminatory power in human participant studies.

Frequency Bands	Measurement Period		
	After Period 3	After Period 4	After Rehydration
F1	$1.67\times 10^{-8}$	$1.00\times 10^{-3}$	$9.69\times 10^{-9}$
F2	$1.40\times 10^{-3}$	$4.40\times 10^{-4}$	$8.68\times 10^{-6}$
F3	$5.00\times 10^{-9}$	$5.70\times 10^{-3}$	$2.34\times 10^{-10}$
F4	$2.20\times 10^{-3}$	$2.70\times 10^{-3}$	$9.74\times 10^{-7}$

**Table 4:** Test decisions and *p*-values of two-sample *t*-tests for the selection of the parameter that best characterizes hydration in human body (a test decision value of 0 indicates a failure to reject the null hypothesis at 95% confidence level and a value of 1 indicates rejection of the null hypothesis at 95% confidence level).

Sr. No.	Parameter	Measurement Time					
		After Period 3		After Period 4		After Rehydration	
		Test Decision	*p*-Value	Test Decision	*p*-Value	Test Decision	*p*-Value
1	Percentage change in Body Weight ($\Delta w_{t}$)	1	$1.06\times 10^{-9}$	1	$4.48\times 10^{-6}$	1	$9.33\times 10^{-4}$
2	Percentage change in Plasma Volume ($\Delta PV_{t}$)	0	$4.10\times 10^{-1}$	0	$1.90\times 10^{-1}$	1	$9.50\times 10^{-3}$
3	Percentage change in Urine Specific Gravity ($\Delta USG_{t}$)	NA	NA	0	$1.30\times 10^{-1}$	1	$3.14\times 10^{-4}$

**Table 5:** Correlation coefficients corresponding to different predictor variables during all measurement times. $\Delta \bar{Fx_{t}}$ denotes the percentage change in the mean of S21 magnitudes corresponding to frequency band *x* at measurement time *t*, as compared to baseline. $\Delta \bar{Fx_{t}}$ is calculated as shown in Equation (6).

Sr. No.	Predictor Variable	Measurement Time			
		After Period 3	After Period 4	After Exercise	After Rehydration
1	$\Delta \bar{F1_{t}}$	−0.26	−0.07	−0.17	0.12
2	$\Delta \bar{F2_{t}}$	−0.11	−0.06	−0.04	0.21
3	$\Delta \bar{F3_{t}}$	−0.30	−0.09	−0.17	−0.02
4	$\Delta \bar{F4_{t}}$	−0.24	−0.14	−0.20	0.08

**Table 6:** Results for general analysis—Case 1: building separate regression models for dehydration and rehydration phases (RM-I: regression models built using $\Delta \bar{F1_{t}}$, $\Delta \bar{F2_{t}}$, $\Delta \bar{F3_{t}}$, and $\Delta \bar{F4_{t}}$ as predictors; RM-II: regression models built using $\Delta \bar{PV_{t}}$ and $\Delta \bar{USG_{t}}$ as predictors).

Sr. No.	Regression Method	Metric	Phase			
			Dehydration		Rehydration	
			RM-I	RM-II	RM-I	RM-II
1	Linear Regression (LR)	MSE	0.26	0.33	0.21	0.28
		Ordinary R^2^	0.22	0.13	0.37	0.16
		Adjusted R^2^	0.18	0.11	0.32	0.15
		Predictive Performance	58.55%	46.38%	22.10%	21.56%
2	Decision Tree Regression (DTR)	MSE	0.14	0.21	0.15	0.19
		Ordinary R^2^	0.52	0.44	0.49	0.41
		Adjusted R^2^	0.49	0.43	0.35	0.35
		Predictive Performance	69.31%	57.21%	39.13%	28.63%
3	Support Vector Regression (SVR)	MSE	0.04	0.11	0.03	0.06
		Ordinary R^2^	0.83	0.71	0.85	0.78
		Adjusted R^2^	0.82	0.67	0.83	0.75
		Predictive Performance	88.55%	78.47%	87.30%	75.39%

**Table 7:** Results for general analysis—Case 2: building a common model for both dehydration and rehydration phases (RM-I: regression models built using $\Delta \bar{F1_{t}}$, $\Delta \bar{F2_{t}}$, $\Delta \bar{F3_{t}}$, and $\Delta \bar{F4_{t}}$ as predictors; RM-II: regression models built using $\Delta \bar{PV_{t}}$ and $\Delta \bar{USG_{t}}$ as predictors).

Sr. No.	Regression Method	Metric	Model Specification	
			RM-I	RM-II
1	Linear Regression (LR)	MSE	0.32	0.38
		Ordinary R^2^	0.19	0.13
		Adjusted R^2^	0.16	0.11
		Predictive Performance	34.26%	26.87%
2	Decision Tree Regression (DTR)	MSE	0.17	0.24
		Ordinary R^2^	0.53	0.39
		Adjusted R^2^	0.51	0.35
		Predictive Performance	56.99%	45.33%
3	Support Vector Regression (SVR)	MSE	0.10	0.12
		Ordinary R^2^	0.73	0.61
		Adjusted R^2^	0.72	0.58
		Predictive Performance	76.32%	70.14%

**Table 8:** Results for sex-specific analysis—Case 1: using the models originally trained on the entire data (both male and female participants).

Sr. No.	Regression Method	Metric	Phase			
			Dehydration		Rehydration	
			Male	Female	Male	Female
1	Linear Regression (LR)	MSE	0.26	0.26	0.19	0.20
		Ordinary R^2^	0.18	0.15	0.47	0.15
		Adjusted R^2^	0.08	0.07	0.32	0.09
		Predictive Performance	65.52%	50.96%	41.56%	32.47%
2	Decision Tree Regression (DTR)	MSE	0.13	0.16	0.25	0.11
		Ordinary R^2^	0.51	0.47	0.29	0.44
		Adjusted R^2^	0.44	0.41	0.19	0.33
		Predictive Performance	77.76%	58.73%	34.10%	42.24%
3	Support Vector Regression (SVR)	MSE	0.06	0.03	0.03	0.02
		Ordinary R^2^	0.75	0.87	0.82	0.85
		Adjusted R^2^	0.72	0.86	0.77	0.82
		Predictive Performance	88.02%	88.74%	85.34%	89.89%

**Table 9:** Results for sex-specific analysis—Case 2: training separate models using data from male and female participants.

Sr. No.	Regression Method	Metric	Phase			
			Dehydration		Rehydration	
			Male	Female	Male	Female
1	Linear Regression (LR)	MSE	0.12	0.18	0.17	0.13
		Ordinary R^2^	0.64	0.44	0.52	0.50
		Adjusted R^2^	0.59	0.38	0.38	0.39
		Predictive Performance	81.35%	59.24%	48.91%	34.67%
2	Decision Tree Regression (DTR)	MSE	0.15	0.18	0.35	0.17
		Ordinary R^2^	0.40	0.40	0.18	0.18
		Adjusted R^2^	0.33	0.35	0.08	0.09
		Predictive Performance	76.62%	55.70%	11.97%	17.50%
3	Support Vector Regression (SVR)	MSE	0.03	0.04	0.03	0.03
		Ordinary R^2^	0.88	0.86	0.87	0.88
		Adjusted R^2^	0.86	0.85	0.83	0.86
		Predictive Performance	91.78%	90.57%	87.28%	90.02%

## Data Availability

Data are available from the authors on a reasonable request.

## Abbreviations

The following abbreviations are used in this manuscript:

THz	Terahertz
GSR	Galvanic Skin Response
SVM	Support Vector Machines
PPG	Photoplethysmographic
FBM	Fluid Balance Measurements
BL	Baseline
NBW	Nude Body Weight
USG	Urine Specific Gravity
HCT	Hematocrit
PV	Plasma Volume
LR	Linear Regression
DTR	Decision Tree Regression
SVR	Support Vector Regression
MSE	Mean Squared Error
